# Prediction of success at UK Specialty Board Examinations using the mandatory postgraduate UK surgical examination

**DOI:** 10.1002/bjs5.50212

**Published:** 2019-09-30

**Authors:** D. S. G. Scrimgeour, J. Cleland, A. J. Lee, P. A. Brennan

**Affiliations:** ^1^ Centre for Healthcare Education Research and Innovation University of Aberdeen Aberdeen UK; ^2^ Department of Medical Statistics University of Aberdeen Aberdeen UK; ^3^ Department of Colorectal Surgery Aberdeen Royal Infirmary Aberdeen UK; ^4^ Intercollegiate Committee for Basic Surgical Examinations

## Abstract

**Background:**

While performance in other mandatory examinations taken at the beginning of a doctor's career are predictive of final training outcomes, the influence early postgraduate surgical examinations might have on success at Specialty Board Exams in the UK is currently unknown. The aim was to investigate whether performance at the mandatory Membership of the Royal College of Surgeons (MRCS) examination, and other variables, are predictive of success at the Fellowship of the Royal College of Surgeons (FRCS) examination, thus potentially identifying those who may benefit from early academic intervention.

**Methods:**

Pearson correlation coefficients examined the linear relationship between both examinations and logistic regression analysis identified potential independent predictors of FRCS success. All UK medical graduates who attempted either section of FRCS (Sections 1 and 2) between 2012 and 2018 were included.

**Results:**

First attempt pass rates for Sections 1 and 2 FRCS were 87.4 per cent (*n* = 854) and 91.8 per cent (*n* = 797) respectively. In logistic regression analysis, sex (male: odds ratio (OR) 2.32, 95 per cent c.i 1.43 to 3.76), age (less than 29 years at graduation: OR 3.22, 1.88 to 5.51), Part B MRCS attempts (1 attempt: OR 1.77, 1.08 to 3.00), Part A score (OR 1.14, 1.09 to 1.89) and Part B score (OR 1.06, 1.03 to 1.09) were independent predictors of Section 1 FRCS success. Predictors of Section 2 FRCS success were age (less than 29 years at graduation: OR 3.55, 2.00 to 6.39), Part A score (OR 1.06, 1.02 to 1.11) and Section 1 FRCS score (OR 1.13, 1.07 to 1.18).

**Conclusion:**

Part A and B MRCS performance were independent predictors of FRCS success, providing further evidence to support the predictive validity of this mandatory postgraduate exam. However, future research must explore the reasons between the attainment gaps observed for different groups of doctors.

## Graphical abstract

We aimed to investigate whether MRCS and other variables are predictive of FRCS success. Performance at Part A and B MRCS were found to be independent predictors of FRCS success, providing further evidence to support the predictive validity of this mandatory postgraduate exam.

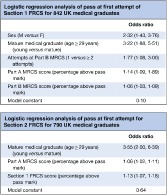

Passing first time predicts future success

## Abstract (es)

**Antecedentes:**

Si bien el resultado obtenido en otros exámenes obligatorios efectuados al inicio de la formación médica son predictores de los resultados finales obtenidos al terminar la etapa de formación, se desconoce la influencia que podrían tener los resultados de primeros exámenes quirúrgicos de postgrado en el éxito de los exámenes de la Comisión de Especialidad del Reino Unido. El objetivo fue investigar si los resultados obtenidos en el examen obligatorio para ser miembro del Real Colegio de Cirujanos (*membership of the Royal College of Surgeons*, MRCS) y de otras variables son predictores de éxito en el examen de ingreso de licenciados universitarios del Real Colegio de Cirujanos (*Fellowship of the Royal College of Surgeons*, FRCS), de modo, que fuera posible identificar a aquellos colectivos de cirujanos que podrían beneficiarse de una acción formativa temprana.

**Métodos:**

Se utilizaron los coeficientes de correlación de Pearson para evaluar la relación lineal entre ambos exámenes, y un análisis de regresión logística para identificar posibles variables predictivas de éxito del examen del FRCS. Se incluyeron todos los graduados médicos del Reino Unido que intentaron cualquier sección del FRCS (secciones 1 y 2) entre los años 2012 y 2018.

**Resultados:**

Al primer intento, las tasas de superación de las secciones 1 y 2 del FRCS fueron del 87,4% (*n* = 854) y del 91,8% (*n* = 797), respectivamente. En el análisis de regresión logística, el sexo (masculino, razón de oportunidades, *odds ratio*, OR 2,32, i.c. del 95% 1,43‐3,76), la edad (menos de 29 años en el momento de la graduación, OR 3,22, 1,88‐5,51), el número de intentos para superar la Parte B del MRCS (un intento, OR 1,77, 1,08‐3,00) y la puntuación obtenida en las Partes A (OR 1,14, 1,09‐1,89) y B del MRCS (OR 1,06, 1,03‐1,09), fueron predictores independientes de éxito en la Sección 1 del FRCS. Los factores predictivos del éxito en la Sección 2 del FRCS fueron la edad (menos de 29 años a la graduación, 3,55, 1,88‐5,51), la puntuación de la Parte A del MRCS (1,06, 1,02‐1,11) y la puntuación de la Sección 1 del FRCS (1,13, 1,07‐1,18).

**Conclusión:**

Los resultados de las partes A y B del MRCS fueron factores predictivos independientes del éxito del FRCS, lo que supone una evidencia adicional en favor de la validez predictiva de este examen obligatorio de postgrado. Sin embargo, la investigación futura debe explorar las razones entre las diferencias de logros observadas para diferentes grupos de médicos.

## Introduction

Irrespective of which specific training pathway they are following^1^, UK surgical trainees must pass a postgraduate surgical examination, the Intercollegiate Membership of the Royal College of Surgeons (MRCS), as well as gain essential competencies to progress into their third year of training.

The MRCS is one of the most widely offered postgraduate surgical exams in the world, with over 6000 UK and overseas doctors taking it each year^2^, ^3^. MRCS comprises two parts (A and B). Part A MRCS is a 5‐h multiple choice question (MCQ) exam and Part B MRCS is an objective structured clinical exam (OSCE) with 18 manned, 9‐min long, stations^4^.

Until recently, and unlike other postgraduate medical examinations in the UK and around the world, there had been no formal analysis of the predictive validity of the MRCS. Thus, whether performance at this examination predicts outcomes as a surgeon in training or beyond was not known. If the MRCS does not predict future clinical and training outcomes, its use as a gatekeeper for progression through surgical training would be inappropriate.

Research to date has found that Part A MRCS performance predicts performance at Part B, and that Part B MRCS performance itself predicts surgical training outcomes^3^, ^5^, ^6^. For example, score and number of attempts at Part B MRCS were found to be significant independent predictors of both selection into higher surgical training (HST, year 3 onwards of training) and performance during HST^3^, ^6^. Similar relationships have been observed between the written papers and clinical exams of the equivalent physician examination in the UK, the Membership of the Royal College of Physicians (MRCP), and for the Medical Licensing Examination® (USMLE®) in the USA^7^, ^8^. Performance at the USMLE® has also been found consistently to predict performance in the American Board of Surgery Qualifying and Certifying Examinations^9^, ^10^, and may explain why US surgical training directors rank USMLE® performance as one of the most important factors in selecting applicants for surgical training^11^.

Towards the end of UK surgical training, trainees attempt the Intercollegiate Specialty Board Examinations. These competency‐based exams are designed to ensure that trainees have attained the standard required of a newly appointed consultant in their chosen surgical specialty.

All surgical Specialty Board Examinations consist of two parts. Like Part A MRCS, Section 1 is a two‐part MCQ‐based exam that must be passed before progressing to Section 2, which comprises patient‐based clinical examinations and scenario‐based structured interviews. Success at Section 2 entitles the candidate to apply to become a Fellow of their nominated surgical College and obtain the rights to use the prestigious postnominal letters FRCS (Fellowship of the Royal College of Surgeons of either England, Edinburgh, Ireland or Glasgow)^12^.

It is clear from the literature that performance in other mandatory examinations taken at the beginning of a doctor's career are predictive of final training outcomes and beyond^9^, ^10^, ^13^, ^14^, ^15^, ^16^, but the influence that early postgraduate surgical examinations may have on Specialty Board Examinations in the UK is unknown. The aim of this study was to investigate whether MRCS predicts first‐attempt pass rate at Sections 1 and 2 of the UK Specialty Board Examinations (FRCS). This will determine whether doctors who perform poorly at the MRCS are at greater risk of failing the FRCS, thereby identifying those who may benefit from early academic intervention^17^, ^18^.

## Methods

The study population included all UK medical graduates who had passed both parts of the MRCS (Parts A and B) and had attempted Section 1 or Sections 1 and 2 of the FRCS since September 2007.

The FRCS database, held by the Joint Committee on Intercollegiate Examinations (JCIE), was cross‐linked with a previously created MRCS database^5^ by an administrative member of the JCIE team. This created a complete MRCS and FRCS examination history for each candidate. These data were cross‐checked and anonymized before being released to the research team. Both first and final attempt scores at each part of the MRCS and FRCS were retrieved for all candidates. Number of attempts at each part of the MRCS and FRCS, date of graduation, date of exam, date of birth and the self‐classified demographics of sex, first language and ethnicity were also recorded.

All variables were dichotomized, other than MRCS and FRCS scores. Self‐declared ethnicity was coded as ‘white’ or ‘non‐white’, self‐declared first language was categorized as ‘English’ or ‘not English’, and number of attempts at each part of the MRCS and FRCS were grouped as ‘one attempt’ or ‘two or more attempts’.

Age at graduation was included, as older doctors (defined in previous studies as age 29 years or more at graduation from medical school)^3^, ^5^, ^6^ have been found to be more likely to have problems progressing through training in the UK^19^.

As pass marks vary from each exam sitting, performance at each part of the MRCS and FRCS were described in terms of percentage relative to the pass mark (a candidate scoring 0 per cent achieved the minimum pass mark for that exam sitting).

There is no ethics committee for either the MRCS or the FRCS, but the Intercollegiate Committee for Basic Surgical Examinations and its Internal Quality Assurance subcommittee, and the JCIE approved the study.

### Statistical analysis

All analyses were conducted using SPSS® v25.0 (IBM, Armonk, New York, USA). Pearson correlation coefficients were used to examine the linear relationship between scores at each part of the MRCS and both Section 1 and Section 2 of the FRCS. Cohen's guidelines^20^ defined the magnitude of correlation between examination scores (*r* = 0·01–0·29, weak correlation; *r* = 0·30–0·49, moderate correlation; *r* ≥ 0·50, strong correlation). Examination pass rates between factors were examined with the χ^2^ test. A complete candidate analysis was also performed using the χ^2^ test to compare pass rates between candidates after inclusion and exclusion of missing first language and ethnicity data. To maximize numbers and improve the sensitivity of the logistic regression analyses, missing data were included as a separate group for both first language and ethnicity variables.

Logistic regression analysis was used to identify any potential independent predictors of pass at first attempt at Sections 1 and 2 of the FRCS. All potential predictors with *P* < 0·100 in univariable analysis were entered simultaneously into the regression models. Any variable with *P* > 0·050 in the full model was subsequently removed until only statistically significant predictors remained in each model. Potential interactions between any of the variables in the final logistic regression models were examined.

## Results

### Section 1 FRCS

Between June 2012 and June 2018, a total of 854 UK medical graduates made 988 attempts at Section 1 of the FRCS. Some 87·4 per cent of UK candidates passed Section 1 FRCS at their first attempt (746 of 854). The majority of candidates were white (449 of 703, 63·9 per cent), men (660 of 850, 77·6 per cent), spoke English as their first language (691 of 735, 94·0 per cent) and had graduated from medical school before the age of 29 years (734 of 846, 86·8 per cent). Most candidates had passed Part A (719 of 854, 84·2 per cent) and Part B (686 of 854, 80·3 per cent) MRCS at the first attempt.

Pass rates for Section 1 FRCS by sex, first language, age, ethnicity and number of attempts at Part A and Part B MRCS are shown in *Table* 1. Pass rates were significantly higher in men (*P* < 0·001), younger graduates (*P* < 0·001), white candidates (*P* = 0·028), and those who had made one attempt at Part A MRCS (*P* < 0·001) and Part B MRCS (*P* < 0·001). Once those with missing first language or ethnicity were included as a separate category to maximize numbers, the pass rate for the missing group was similar to that of the English and white groups respectively.

**Table 1:** First‐attempt pass rates at Sections 1 and 2 of the FRCS by sociodemographics (sex, first language, age and ethnicity), number of attempts at Parts A and B of the MRCS, and number of attempts at Section 1 FRCS for UK medical graduates

	First‐attempt pass rate (%)	
	Section 1 (*n* = 854)	Section 2 (*n* = 797)
**Sex**		
M	90·2 (595 of 660)	92·5 (577 of 624)
F	77·9 (148 of 190)	89·3 (151 of 169)
Missing data	4	4
*P* *	< 0·001	0·190
**First language**		
English	87·7 (606 of 691)	93·0 (601 of 646)
Not English	82 (36 of 44)	79 (33 of 42)
Missing data	87·4 (104 of 119)	89·9 (98 of 109)
*P* *	0·523	0·003
**Mature medical graduate (≥ 29 years old at graduation)**		
No	89·9 (660 of 734)	94·2 (647 of 687)
Yes	70·5 (79 of 112)	75·7 (78 of 103)
Missing	8	7
*P* *	< 0·001	< 0·001
**Ethnicity**		
White	89·3 (401 of 449)	94·3 (396 of 420)
Non‐white	82·7 (210 of 254)	87·7 (207 of 236)
Missing	89·4 (135 of 151)	91·5 (129 of 141)
*P* *	0·028	0·013
**Attempts at Part A MRCS**		
1	90·1 (648 of 719)	93·5 (638 of 682)
≥ 2	72·6 (98 of 135)	81·7 (94 of 115)
*P* *	< 0·001	< 0·001
**Attempts at Part B MRCS**		
1	90·4 (620 of 686)	92·6 (601 of 649)
≥ 2	75·0 (126 of 168)	88·5 (131 of 148)
*P* *	< 0·001	0·132
**Attempts at Section 1 FRCS**		
1	–	93·2 (670 of 719)
≥ 2	79·5 (62 of 78)
*P* *		< 0·001

MRCS, Membership of the Royal College of Surgeons examination; FRCS, Fellowship of the Royal College of Surgeons examination.

* χ^2^ test.

*Table* 2 shows the odds ratios (ORs) and 95 per cent c.i. for passing Section 1 FRCS at the first attempt. Men were more than twice as likely to pass Section 1 FRCS compared with women, and younger medical graduates were more than three times as likely to pass Section 1 at first attempt than mature medical graduates. Section 1 FRCS candidates were nearly twice as likely to pass the exam at first attempt if they had also passed Part B MRCS at first attempt.

**Table 2:** Logistic regression analysis of pass at first attempt of Section 1 FRCS for 842 UK medical graduates

	Odds ratio
Sex (M *versus* F)	2·32 (1·43, 3·76)
Mature medical graduate (age ≥ 29 years) (young *versus* mature)	3·22 (1·88, 5·51)
Attempts at Part B MRCS (1 *versus* ≥ 2 attempts)	1·77 (1·08, 3·00)
Part A MRCS score (percentage above pass mark)	1·14 (1·09, 1·89)
Part B MRCS score (percentage above pass mark)	1·06 (1·03, 1·09)
Model constant	0·10

Values in parentheses are 95 per cent confidence intervals. MRCS, Membership of the Royal College of Surgeons examination; FRCS, Fellowship of the Royal College of Surgeons examination.

For every 1 per cent over the pass mark that each candidate had achieved at Part A and Part B MRCS, their chances of passing Section 1 FRCS at first attempt increased by 14 per cent for Part A and 6 per cent for Part B MRCS.

Ethnicity and number of attempts at Part A MRCS were not independent predictors of Section 1 FRCS success (*P* = 0·555 and *P* = 0·111 respectively). There were no statistically significant interactions between any of the Section 1 variables in the final logistic regression model.

### Section 2 FRCS

In total, 797 UK medical graduates made 855 attempts at Section 2 FRCS from September 2012 to September 2018. Some 732 candidates (91·8 per cent) passed Section 2 FRCS at first attempt. Unsurprisingly, the demographics for Section 2 FRCS candidates were similar to those observed for Section 1 (*Table* 1). Most candidates were white, men, spoke English as their first language, and had graduated from medical school before the age of 29 years. The majority of candidates had passed Parts A and B MRCS at the first attempt.

First‐attempt pass rates for Section 2 FRCS by sex, first language, age at graduation, ethnicity, number of attempts at Part A and Part B MRCS, and number of attempts at Section 1 FRCS are shown in *Table* 1. Differences in pass rates were statistically significant for first language (*P* = 0·003), age (*P* < 0·001), ethnicity (*P* = 0·013), and number of attempts at Part A MRCS (*P* < 0·001) and Section 1 FRCS (*P* < 0·001). As for Section 1 FRCS, once those candidates with missing data (first language or ethnicity) were included, the pass rate for the missing groups was similar to that of the English and white groups.

*Table* 3 shows the final logistic regression model for pass at first attempt of Section 2 FRCS. Younger medical graduates were 3·55 times more likely to pass Section 2 FRCS at first attempt compared with mature medical graduates. Part A MRCS and Section 1 FRCS scores were both independent predictors of passing Section 2 FRCS at first attempt. For every percentage point over the pass mark that each candidate achieved for Part A MRCS and Section 1 FRCS, their chances of passing Section 2 FRCS at first attempt increased by 6 per cent for Part A and 13 per cent for Section 1.

**Table 3:** Logistic regression analysis of pass at first attempt for Section 2 FRCS for 790 UK medical graduates

	Odds ratio
Mature medical graduate (age ≥ 29 years) (young *versus* mature)	3·55 (2·00, 6·39)
Part A MRCS score (percentage above pass mark)	1·06 (1·02, 1·11)
Section 1 FRCS score (percentage above pass mark)	1·13 (1·07, 1·18)
Model constant	0·64

Values in parentheses are 95 per cent confidence intervals. MRCS, Membership of the Royal College of Surgeons examination; FRCS, Fellowship of the Royal College of Surgeons examination.

First language, ethnicity, and number of attempts at both Part B MRCS and Section 1 FRCS were not found to be independent predictors of Section 2 FRCS success. There were no statistically significant interactions between any of the Section 2 FRCS variables in the final model.

### Relationship between Part A and B MRCS scores and Section 1 and 2 FRCS scores

A strong positive correlation was found between Part A MRCS score and Section 1 FRCS first‐attempt score (*Table* 4). A moderate positive correlation was found for Part A MRCS score and Section 2 FRCS first‐attempt score. There was a weak correlation between Part B MRCS and first‐attempt score at Section 1 FRCS and a moderate correlation between Part B MRCS score and Section 2 FRCS first‐attempt score. A significant, moderate positive correlation was found between Section 1 FRCS score and Section 2 FRCS first‐attempt score. All correlations were significant at *P* < 0·001.

**Table 4:** Correlations between Part A and B MRCS scores and Section 1 and 2 FRCS scores

	Section 1 FRCS (*n* = 854)	Section 2 FRCS (*n* = 797)
Part A MRCS	0·50	0·34
Part B MRCS	0·29	0·34
Section 1 FRCS	–	0·45

MRCS, Membership of the Royal College of Surgeons examination; FRCS, Fellowship of the Royal College of Surgeons examination. All correlations were significant at *P* < 0·001.

## Discussion

This is the first study to report on the relationship between mandatory early postgraduate surgical examinations and UK Surgical Specialty Board Examinations. The high first‐attempt pass rates observed in this study for Sections 1 and 2 of the FRCS (87·4 and 91·8 per cent respectively) are reassuring both to surgical trainees approaching their exam and to the general public, as they suggest that the vast majority of UK medical graduates are sufficiently prepared at the end of surgical training.

Performance at Part A and Part B MRCS predicted FRCS performance, but only the Part A score predicted both Section 1 and Section 2 FRCS first‐attempt success. For every 1 per cent over the pass mark that each trainee achieved at the written knowledge‐based Part A MRCS, their chances of passing Sections 1 and 2 FRCS increased by 14 and 6 per cent respectively. Similar relationships have been observed between USMLE® Step 1 performance and success on the American Board of Certifying Examinations in the USA^9^. It has also been found previously that Part B MRCS score is predictive of both selection to HST and performance during HST in the UK^3^, ^6^. In the present study, it was observed that the Part B MRCS score could predict Section 1 FRCS success, with the probability of passing Section 1 increasing by 6 per cent for every 1 per cent increment in Part B score (percentage over the pass mark). In addition to Part B score, it has also been identified previously that the number of attempts required to pass this examination is predictive of these training outcomes^3^, ^6^.

Attempts at Part B MRCS were predictive of success at Section 1 FRCS, with candidates almost twice as likely to fail Section 1 if they had failed Part B MRCS at first attempt. This is of interest as, although trainees often take several attempts to pass early postgraduate examinations^7^, ^21^, most studies have focused on the association between examination score and future performance, without considering number of attempts needed to pass. However, when number of attempts is taken into consideration, a clear inverse relationship between attempts and other performance indicators exists. This pattern is also seen in other medical education assessments. For example, USMLE® Step 1 failure is associated with an increased risk of not being specialty board certified^22^, and multiple attempts at the Medical College Admission Test have been found to be associated with an increased risk of failing USMLE® Step 2^23^. Similarly, in the UK, failing the Professional and Linguistic Assessments Board test at first attempt is independently predictive of unsatisfactory training outcomes, compared with those who pass at first attempt^24^. Another study^7^ from the UK found that as the number of attempts at each part of the MRCP increased, the final passing scored decreased. This pattern was also observed for the MRCS^5^.

The present study adds to the evidence that early performance predicts later performance. Trainees who require multiple attempts to pass early postgraduate examinations are more likely to have problems not only during training but also on completion of training. This suggests that multiple attempts to pass postgraduate examinations could be an indicator to initiate remedial action plans and careers advice.

Interestingly, no significant association was found between number of attempts at Section 1 FRCS and Section 2 FRCS pass rate, although Section 1 FRCS score was an independent predictor of Section 2 success. For every 1 per cent over the Section 1 pass mark, the chances of passing Section 2 increased by 13 per cent.

The relationship between sex and performance on postgraduate examinations remains an enigma. Men outperformed women in Section 1 FRCS, but there was no difference for Section 2 FRCS. These results echo findings from the only other study^25^ that assessed sex influences on FRCS pass rates, and similar observations have also been made for the MRCS and USMLE®^5^, ^26^, ^27^, ^28^. Qualitative research unpacking the possible reasons for this pattern is imperative to ensure that UK surgical examinations are fair and unbiased.

Despite there being a clear relationship between ethnicity and differential attainment in postgraduate examinations, including the MRCS, no such relationship for the FRCS was observed in this study^5^, ^6^, ^25^, ^26^, ^28^. Again, the possible reasons for this merit exploration.

In the UK, older doctors have been found to be more likely to have problems progressing through specialty training and passing early postgraduate examinations^5^, ^19^. A similar pattern has been reported in the USA, with a recent study^28^ finding that older candidates are more likely to obtain significantly lower USMLE® Step 1, 2 and 3 scores than their younger colleagues. In the present study, older doctors were more than twice as likely to fail Section 1 FRCS and nearly four times as likely to fail Section 2. This previously unknown fact warrants further investigation to determine both the reasons behind this association and how older doctors can be supported and advised throughout surgical training.

The exclusion of non‐UK medical graduates from the overall analysis may be considered a limitation of this study. However, it was felt that this exclusion would create the most homogeneous group of candidates attempting FRCS and potentially increase the accuracy of the results. In addition, both MRCS and FRCS have been designed to assess trainees who are in, or have been through, the UK training system, and are therefore more likely to continue their careers in the UK. However, future research might usefully look at the whole population of those who sit both the MRCS and the FRCS to compare examination performance across different groups. Another potential limitation was missing data for self‐declared first language and ethnicity, as a routine data set collected for administrative purposes was used. An attempt was made to obviate this issue by including missing data as a separate group in the analyses.

This is the first study to investigate the relationship between early postgraduate medical examinations and Specialty Board Examinations in the UK. Performance at Part A and B MRCS were found to be independent predictors of FRCS success. This provides further evidence supporting the predictive validity of MRCS and potentially justifies its current role as a mandatory component of the educational curriculum for UK surgical trainees.
